# Bleached, But Not by the Sun: Sunscreen Linked to Coral Damage

**DOI:** 10.1289/ehp.116-a173b

**Published:** 2008-04

**Authors:** John Tibbetts

Warm, shallow, sun-drenched seas sparkling with brilliantly colored fish and coral species—we’ve all seen dazzling images of tropical reefs. Coral reefs are among the most biologically productive and diverse ecosystems in the world, providing food protein for half a billion people. But tropical reefs have begun dying from bleaching, with the frequency and spatial extent of such bleaching increasing dramatically over the past 20 years. Now a study finds that chemical compounds in sunscreen products can cause abrupt and complete bleaching of hard corals, even at extremely low concentrations **[*EHP* 116:441–447; Danovaro et al.]**.

Zooxanthellae, symbiotic algae that live in healthy coral tissue, provide nutrients to corals through photosynthesis. The algae also help make the spectacular colors for which corals are known. The corals lose their color when zooxanthellae die or leave the reef; the protective skeletons of the corals are thus exposed, and the corals die. Rising seawater temperatures, bacterial and viral diseases, ultraviolet light or other radiation, and pollution have been blamed for coral bleaching.

Scientists at the Polytechnic University of the Marche Region in Ancona, Italy, studied the effects of sunscreen exposure on samples from tropical reefs. The researchers collected branches of hard coral from sites in the Red Sea, the Caribbean Sea, the Indian Ocean off Thailand, and the Pacific Ocean near Indonesia.

Coral branches were immersed in bags of virus-free seawater supplemented with various quantities of sunscreen, then incubated *in situ*. These samples were compared with controls also incubated *in situ* in virus-free seawater.

The researchers found that among the several brands of sunscreen tested, four commonly found ingredients—paraben, cinnamate, benzophenone, and camphor derivatives—can stimulate dormant viral infections in zooxanthellae. The sunscreen chemicals caused viruses within zooxanthellae to replicate until their algal hosts exploded, spilling viruses into the surrounding seawater, which could then spread infection to nearby coral communities.

Coral bleaching occurred, often within a few hours, but always within 4 days, at sunscreen quantities as small as 10 μL/L. Controls remained healthy.

The researchers estimate that approximately 10% of the world’s coral reefs are potentially threatened by sunscreen that washes off swimmers in reef waters. The study suggests that, as tourism continues to increase in tropical reef areas, the impact of sunscreens on coral bleaching could rise significantly in the future.

## Figures and Tables

**Figure:**
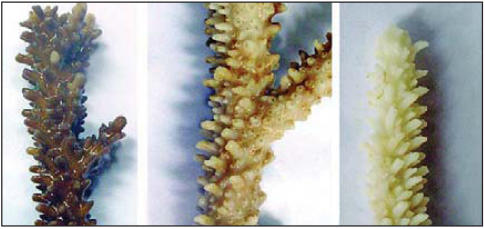
Higher temperatures worsen bleaching (left to right) Control sample of *Acropora divaricata*; exposed sample incubated at 28°C; exposed sample incubated at 30°C.

